# Signaling lymphocytic activation molecules Slam and cancers: friends or foes?

**DOI:** 10.18632/oncotarget.24575

**Published:** 2018-02-26

**Authors:** Gregory Fouquet, Ingrid Marcq, Véronique Debuysscher, Jagadeesh Bayry, Amrathlal Rabbind Singh, Abderrahmane Bengrine, Eric Nguyen-Khac, Mickael Naassila, Hicham Bouhlal

**Affiliations:** ^1^ INSERM 1247-GRAP, Centre Universitaire de Recherche en Santé CURS, Université de Picardie Jules Verne, CHU Sud, Amiens, France; ^2^ INSERM UMRS 1138, Centre de Recherche des Cordeliers–Paris, Paris, France; ^3^ Department of Microbiology, Aravind Medical Research Foundation, Anna Nagar, Madurai-India; ^4^ Biobanque de Picardie, Centre Hospitalier Universitaire Sud, Amiens, France; ^5^ Service Hepato-Gastroenterologie, Centre Hospitalier Universitaire Sud, Amiens, France

**Keywords:** SLAMF molecules, cancer, pathophysiology, therapy

## Abstract

Signaling Lymphocytic Activation Molecules (SLAM) family receptors are initially described in immune cells. These receptors recruit both activating and inhibitory SH2 domain containing proteins through their Immunoreceptor Tyrosine based Switch Motifs (ITSMs). Accumulating evidence suggest that the members of this family are intimately involved in different physiological and pathophysiological events such as regulation of immune responses and entry pathways of certain viruses. Recently, other functions of SLAM, principally in the pathophysiology of neoplastic transformations have also been deciphered. These new findings may prompt SLAM to be considered as new tumor markers, diagnostic tools or potential therapeutic targets for controlling the tumor progression. In this review, we summarize the major observations describing the implications and features of SLAM in oncology and discuss the therapeutic potential attributed to these molecules.

## INTRODUCTION

### Molecular characteristics of SLAM family receptors

SLAM family receptors belong to the Immunoglobulin (Ig) superfamily. The SLAM family contains nine members that possess an extracellular segment comprising two or four Ig-like domains (V-like variable and C2-like constant), a transmembrane segment and a cytoplasmic tail. All members of type-1 transmembrane glycoproteins with the exception of SLAMF2, link to the cell membrane through glycosyl-phosphatidylinositol (GPI) anchor [1, 2]. The cytoplasmic portion is characterized by the presence of several tyrosine motifs TxYxxI/V (ITSM), (T is threonine, I is isoleucine, V is valine and X is any amino acid) [3–5]. However, SLAMF2 lacks ITSM domains due to the absence of C-terminal domain [6]. Meanwhile, even though SLAMF8 and SLAMF9 have a short cytoplasmic tail (~30 amino acid residues), they also lack tyrosine motifs [7, 8]. SLAMF3 exhibits splitting of its two extracellular domains [9, 10]. SLAM members are activated mostly by homophilic interactions *via* their V-like N-terminal domains except SLAMF2 and SLAMF4, which are activated by heterophilic interactions [11]. The functional importance of SLAM-related receptors in immune response is highlighted by the identification of molecular defect responsible for X-linked lymphoproliferative (XLP) syndrome [12]. Indeed, the gene mutated in XLP is found to code for a small adapter-like protein named SLAM-associated protein (SAP) or SH2D1A (hereafter termed SAP). SAP is composed of almost entire Src homology 2 (SH2) domain, and binds with high specificity and affinity to tyrosines in the intracellular domain of SLAM-related receptors [12]. In human, upon activation, SLAMF receptors interact with SLAMF associated protein (SAP) and Ewing’s sarcoma’s-Activated Transcript 2 (EAT-2) to form a receptor complex. The SAP family also includes EAT-2 related transducer (ERT, also known as SH2D1W) [13].

The newly formed complex undergoes phosphorylation at tyrosine residues by Fyn tyrosine kinase leading to recruitment of additional effector molecules [14]. Several lines of evidence support the idea that SLAM family members can provide a second signal for the stimulation of immune cells. SLAM/SAP-dependent functions in immune regulation include natural killer (NK) and T-cell development, B-cell regulation and antibody production/isotype switching and NK-cell cytotoxicity [14]. EAT-2, a SLAM-associated adaptor is expressed in innate immune cells such as dendritic cells (DCs), macrophages and NK cells and it facilitates SLAM-dependent expression of pro-inflammatory cytokines in these cells [6]. Like other members of SLAM family, SLAMF3 recruits SAP and EAT-2 *via* its SH2 domain [15] with the exception that SLAMF3 is the only member, which is able to interact with μ2 sub-unit of AP-2 complex through its Y^470^ motif [16]. The SLAMF3-AP-2 interaction is essential for endocytosis of this complex in immune cells. Upon endocytosis, in T cells, 70 to 80% of SLAMF3 receptors are degraded in the lysosomal compartment, while others are recycled to the surface. In contrast, majority of the receptors are degraded upon internalization in B cells [16]. The internalization of receptor is also regulated by TCR- and BCR-mediated signaling, which, enhance the rate of endocytosis. Thus, endocytosis of the receptor represents an essential mechanism of modulation of surface expression of SLAMF3.

**Figure 1:**
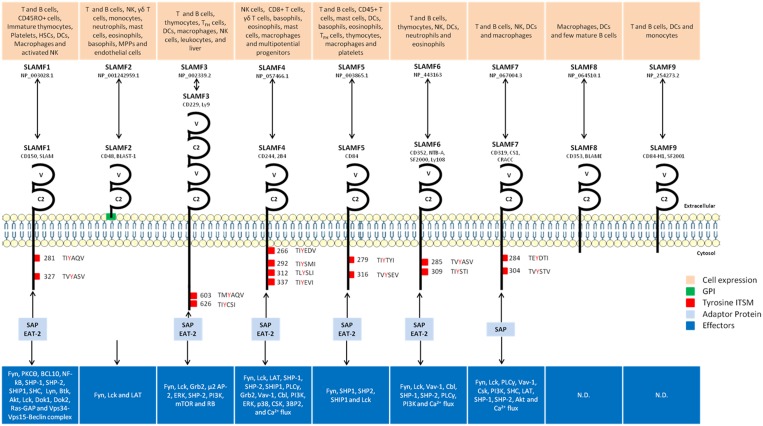
SLAM members, cellular and molecular characteristics HSCs: Hematopoietic Stem Cells; DCs: Dendritic Cells; NK: Natural Killer; PKCƟ: Protein Kinase C Ɵ; BCL10: B-Cell Lymphoma 10; NF-ƙB: Nuclear Factor-ƙB; SHP-1/2: SH2 domain-containing Phosphatase 1/2; SHIP1: SH2-containing Inositol 5'-polyphosphatase 1; SHC: Src Homology 2 domain Containing; Btk: Bruton’s tyrosine kinase; Lck: Lymphocyte-specific protein tyrosine kinase; Dok1/2: Docking protein 1/2; Ras-GAP: Ras GTPase-activating proteins; LAT: Linker for activation of T cells; Grb2: Growth factor receptor bound protein 2; AP-2: Adaptor Protein complex-2; ERK: Extracellular signal-Regulated Kinases; PI3K: PhosphoInositide 3-Kinase; mTOR: mammalian Target of Rapamycin; RB: Rétinoblastoma; PLCγ: PhosphoLipase Cγ; Cbl: Casitas B-lineage Lymphoma; CSK: COOH-terminal Src kinase; 3BP2: Abl-SH3 Binding Protein 2. Localisation of ITSM (TxYxxI/V) were determined on Ensembl. [1, 2, 11, 21, 84, 95].

SLAMF3 is the only member of SLAM family, which has ability to bind directly to Grb2. This adaptor protein is known to activate Ras-MAPK signaling pathway through the recruitment of Son of Sevenless molecule (SOS) [17]. In T cells, Grb2-SH2 domain binds to SLAMF3 phosphorylated at Y^606^ residue. SLAMF3 phosphorylation is performed by Fyn or Lck [18]. Moreover, Grb2-binding site is required for the receptor internalization in T cells following commitment of SLAMF3 or TCR. The co-ligation of SLAMF3 and TCR inhibits ERK phosphorylation as well as cytokine production as opposed to co-ligation of TCR with other members of the SLAM family. It is worth to mention that Grb2-binding site (Y^606^) is different from those of SAP (Y^603^ and Y^626^) and AP-2 (Y^470^) [18–20] (Figure 1).

**Figure 2:**
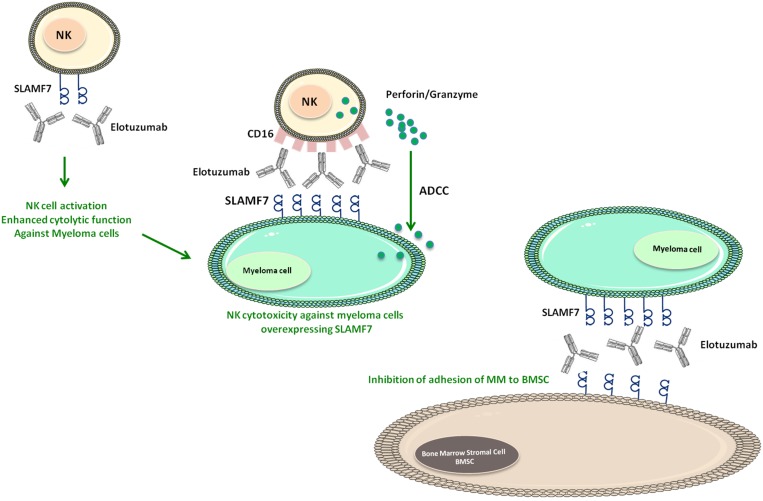
Mechanisms of Elotuzumab against myeloma cells SLAMF7 and CD16 on NK cells can bound to Elotuzumab. The binding to SLAMF7 induces NK cell activation and enhances cytolysis function against myeloma cells. While the binding to CD16 receptor can induce the release of Perforin/Granzyme by NK cells and subsequently enhance ADCC against myeloma cells overexpressing SLAMF7. Moreover, CD16 (NK cells), Elotuzumab and SLAMF7 (Myeloma cells) can enhance NK cell cytotoxicity against myeloma cells independent of ADCC. Furthermore, Elotuzumab-SLAMF7 interaction can inhibit the adhesion between myeloma cells and BMSC.

### SLAM members in hematopathologies

Members of SLAM family are known to be implicated in the pathophysiology of hematologic complications. For this reason, some of them are targets for the monoclonal antibody therapies that are being tested in different clinical trials. In this section, we summarize the implication of SLAM receptors in hematopathologies with an emphasis on their utility in diagnosis and as therapeutic targets (Table 1).

**Table 1:** SLAM members implicated in hematological affections and targeted by diagnosis and therapeutic strategies

SLAM Family	Cancer	Expression	References
SLAMF1	MM	↘	[23]
	CLL	↘	[24]
	ALL	Unmodified	[27]
	B lymphoma	↗	[28]
	Hodgkin’s lymphoma	↗	[30]
	Non-Hodgkin’s Lymphoma	Unmodified	[29]
SLAMF2	CLL	Unmodified	[35]
	B lymphoma	Unmodified	[36]
	MM	↗	[37]
SLAMF3	B-CLL	↗	[49]
	MM	↗	[50]
SLAMF4	AML	Unmodified	[55]
SLAMF5	CLL	↗	[62]
SLAMF6	CLL	↗	[67]
	Lymphoma	↗	[67]
SLAMF7	MM	↗	[70]

Abbreviations: MM: Multiple Myeloma; CLL: Chronic Lymphocytic Leukemia; ALL: Acute Lymphocytic Leukemia; AML: Acute Myeloid Leukemia.

### SLAMF1

SLAMF1 (CD150) is the prototype member of SLAM family that initiates signal transduction networks in T lymphocytes, NK and antigen-presenting cells [6, 14]. During B-T lymphocytes cooperation, SLAMF1 acts as a self-ligand to initiate a signal transduction pathway and to increase lymphocyte activation. SLAMF1 is highly expressed by thymocytes (highest by double positive cells), T and B lymphocytes (overexpressed upon activation), DC, platelets, hematopoietic stem cells (HSCs) and macrophages. Of note, overexpression of SLAMF1 has been reported in monocytes and macrophages of patients with Crohn’s disease and in T lymphocytes from rheumatoid arthritis patients [21, 22].

SLAMF1, in addition to CD86 and CD200, were identified as robust markers that could be added to a routine panel for plasma cell (PC) identification and minimal residual disease evaluation in multiple myeloma (MM) [23]. SLAMF1 expression is also decreased in patients with aggressive chronic lymphocytic leukemia (CLL) and was associated with reduced overall survival [24] (Table 1). Therefore, the loss of SLAMF1 expression (SLAMF1^low^) could indicate an aggressive form of CLL and hence could help in improving patient management as predictive factor of overall survival (OS). *Ex vivo*, the SLAMF1 ligation with stimulating monoclonal antibody in CLL cells, induces phosphorylation of p38, JNK1/2 and Bcl-2, and promotes autophagy. On the contrary, cells with silenced-SLAMF1 are resistant to induction of autophagy [24]. Furthermore, SLAMF1 in combination with *SKI* gene (v-SKI avian sarcoma viral oncogene homolog), was also proposed as robust indicator of prognosis in untreated CLL patients [25].

SLAMF1 also acts as receptor for measles virus (MV) [26]. Studies employing MV as an oncolytic virotherapy agent have suggested that SLAMF1 is a therapeutic target in certain hematological diseases. For instance, in mouse models, this therapeutic strategy has been evaluated using attenuated MV to target certain acute lymphoblastic leukemia (ALL) [27]. Attenuated MV efficiently killed leukemia cells without affecting normal human blood cells and progenitors. A few intravenous injections of attenuated MV were able to eradicate leukemic blasts [27]. Takeda et al. reported higher susceptibility of lymphoma cells to attenuated therapeutic MV vaccine strain (CAM-70)-induced cytolysis due to elevated expression of SLAMF1 in these cells. Similarly, using SLAMF1 expression in cancer cells, the MV oncolytic virotherapy could serve as an alternative therapy against Epstein-Barr Virus (EBV)-positive diffuse large B-cell lymphoma [28]. MV OVT might also target SLAMF1 to fight non-Hodgkin’s lymphoma (Table 3). A vaccine containing MV has been used as an oncolytic agent against mantle cell lymphoma, an aggressive, difficult to cure, but radiosensitive B-cell non-Hodgkin’s lymphoma. Using sodium-iodide symporter loaded MV, a high concentration of iodide was achieved within infected tumor cells. The combination of virotherapy and systemic ^(131)^I resulted in rapid disease regression compared to other therapy alone. The SLAMF1-dependent entry of therapeutic MV allows efficient viral spread, tumor regression, and prolonged survival [29] (Table 2).

**Table 2:** SLAM members implicated in anti-cancer clinical trials

SLAM family	Pathology	Treatment type	Treatment name	Study phase	References
SLAMF1	ALL	Oncolytic MV vaccine strains	CAM-70	Preclinical	[28]
	Non-Hodgkin’s lymphoma		MV^VAC2^NIS	Preclinical	[29]
SLAMF2	CLL	anti-SLAMF2 Ab	WM-63	Phase 1 clinical trial	[35]
	B lymphoma		HuLy-m3	Preclinical	[36]
	MM		1B4	Preclinical	[37]
SLAMF4	Leukemia	2B4 chimeric receptor	2B4-ζ	Preclinical	[57]
SLAMF6	CLL and lymphoma	anti-SLAMF6 Ab	994.1 and 480.12	Preclinical	[67]
	CLL		αSLAMF6 and Ibrutinib	Preclinical	[68]
SLAMF7	MM	CS1-specific peptide	CS1_239–247_	Preclinical	[75]
		anti-SLAMF7 Ab	HuLuc63	Preclinical	[70, 72]
			Elotuzumab combined to Bortezomib	Preclinical	[71]
			Elotuzumab combined to Bortezomib	Phase 1 clinical trial	[77]
			Elotuzumab combined to Lenalidomide and Dexamethasone	Phase 1 clinical trial	[79]
			Elotuzumab combined to Lenalidomide and Dexamethasone	Phase 3 clinical trial	(ELOQUENT-1, NCT01335399)
	MM with renal impairment	anti-SLAMF7 Ab	10 mg/Kg Elotuzumab, 5-25 mg Lenalidomide and 40 mg Dexamethasone	Phase 1b clinical trial	[81]
	Refractory or relapsed MM	anti-SLAMF7 Ab	Elotuzumab	Phase 1 clinical trial	[76]
			10 or 20 mg Elotuzumab, 25 mg Lenalidomide and 40 mg Dexamethasone	Phase1b-2 clinical trial	[80]
			10 mg/Kg Elotuzumab, 25 mg Lenalidomide and 40 mg Dexamethasone	Phase 3 clinical trial	[82]
			10 mg/Kg Elotuzumab, 1,3 mg Bortezomib and 20 mg Dexamethasone	Phase 2 clinical trial	[78]
			10 mg/Kg Elotuzumab, 200 mg Thalidomide and 40 mg Dexamethasone	Phase 2 clinical trial	[83]
	Elotuzumab in combination with Lenalidomide and Dexamethasone obtained FDA in November 2015				

Abbreviations: MM: Multiple Myeloma; CLL: Chronic Lymphocytic Leukemia; ALL: Acute Lymphocytic Leukemia.

**Table 3:** SLAM members implicated in solid cancers

SLAM Family	Cancer	Expression	References
SLAMF1	CNS tumors	nCD150	[85]
SLAMF2	HCC	↗	[87]
SLAMF3	HCC	↘	[90]
SLAMF4	HCC	↗	[87]

Abbreviations: HCC: HepatoCellular Carcinoma; CNS: Central Nerve System.

One of the therapeutic strategies used to eliminate cancer cells is induction of apoptosis and/or autophagy. In this scenario, interaction of SLAMF1 expressed on infiltrating T cells with SLAMF1 of Hodgkin’s lymphoma cells inhibits cell proliferation and induces apoptosis in L1236 Hodgkin’s lymphoma cells independent of JNK activity [30]. Because, SLAMF1 regulates phosphorylation of MAPKs Erk1/2 and p38, it has been proposed that it can contribute to the regulation of tumor cell maintenance in low-rate proliferating Hodgkin’s lymphoma cells [30].

In addition to hemophilic interaction of SLAMF1, its ligation to SAP is also suggested to play a crucial role in the activation of Akt signaling [31]. This activation occur in normal tonsillar B cells and Hodgkin’s lymphoma B cells [32]. SLAMF1-mediated phosphorylation of Akt activates the phosphorylation of its downstream targets (GSK-3β and FoxO1) in EBV-transformed and Hodgkin›s lymphoma cells. Thus by triggering one of the mechanisms, SLAMF1 could suppress apoptosis and hence support survival of immortalized B cells [32].

### SLAMF2

SLAMF2 (CD48, B-lymphocyte activation marker BLAST-1) is reported to be expressed in NK, CD8, γδ T cells, basophils, eosinophils, mast cells and multipotent progenitor [21, 33]. SLAMF2 was described as low affinity ligand for human CD2 [34]. One of the SLAMF2 specificities is the heterophilic interaction with its ligand SLAMF4 [11] (Figure 1). Several studies have targeted SLAMF2 in cancer therapies. Murine anti-SLAMF2 IgM antibody WM63 was used in a pilot CLL phase I clinical trial and results showed a transient reduction in the number of circulating cancer cells [35] (Table 2). The potential application of same anti-SLAMF2 IgM antibody was also reported in case of B lymphoma [36]. Since SLAMF2 is highly expressed on more than 90% MM plasma cells compared to normal lymphocytes (Table 1), this receptor serves as target for mAb therapy in MM [37]. Anti-SLAMF2 antibody induces antibody-dependent cell-mediated cytotoxicity and complement-dependent cytotoxicity against MM cells *in vitro*. In severe combined immunodeficient mice, anti-SLAMF2 antibody inhibited tumor growth without damaging normal CD34+ hematopoietic stem/progenitor cells (HSC) [37] (Table 2). SLAMF2 maintains hematopoiesis and HSC CD34+ pool by controlling IFN-γ production, which negatively affects self-renewal. Low level expression of SLAMF2, and lack of efficient SLAMF2-SLAMF4-induced signaling, leads to lowered levels of IFN-γ and more long-term quiescent CD34+ HSCs in the bone marrow [38]. More importantly, during chemotherapy cures, the microenvironment around HSC in the absence or presence of low expression of SLAMF2 and IFN-γ renders quiescent cells refractory to molecules such as 5-FU and decreases its therapeutic efficiency [38]. A major concern regarding the use of SLAMF2 as a therapeutic target is its broad expression in normal lymphocytes and monocytes, which might cause severe cytopenia and immunosuppression when anti-SLAMF2 mAb is used as a therapeutic drug. Therefore, due to hematological toxicities, anti-SLAMF2 mAb might not be suitable for a long-term maintenance therapy. The potential hematological toxicity of anti-SLAMF2 mAb should therefore, be carefully tested at the pre-clinical stage. These data indicate that anti-SLAMF2 mAb may well turn out to be an effective tool for the improvement of MM patient survival [39].

### SLAMF3

SLAMF3 (CD229, Ly9 in mice) was initially described in thymocytes (highest by double negative and single positive cells), T cells, follicular helper T, B cells (increased expression upon activation), DCs, macrophages and NK cells [21] but its function remains unclear. This receptor is recruited to the contact site between T cells and B cells during antigen presentation process and it is involved in the formation of the immunological synapse [40]. The triggering of SLAMF3 on human T cells induces the rapid phosphorylation of its tyrosine residues and decreases the activation of ERK and the production of CD3-induced IFN-γ [18]. Endocytosis of SLAMF3 blocks lymphocyte activation pathways indicating that the expression of SLAMF3 at the cell surface could regulate lymphocyte functions. In mice, stimulation of SLAMF3 decreases the production of IFN-γ, IL-2, IL-4, IL-6, IL-10 and TNF-α by CD3-activated T cells [41]. However, analysis of T cells from SLAMF3 (Ly9-/−)-deficient mice demonstrated the role of SLAMF3 in T cell activation, as well as in the production of IL-2 and Th2-type cytokines [42]. SLAMF3 expression has been recently shown to correlate with the certain autoimmune disorders. SLAMF3 and SLAMF6 co-stimulation increases IL-17A production by Th17 cells and the correlation between their surface expression and disease activity in systemic lupus erythematosus has been reported [43].

One of the conditions for an effective cancer immunotherapy is the identification of tumor-associated antigens (TAAs), which are increased during neoplastic transformation. These antigens have to be processed and presented by major histocompatibility complex molecules allowing recognition by T cells. Studies with peptides derived from human TAAs indicated that most tumors expressing these antigens could be attacked by specific cytotoxic T cells [44–46]. Some antigen presenting cells, such as B-CLL cells, are ineffective antigen presenting cells and poor stimulators of a primary immune response because they are lacking in costimulatory and adhesion molecules [47]. SLAMF3 is highly expressed in B-CLL cells, processed and efficiently presented as TAA [48, 49] (Table 1). Presenting SLAMF3 allows the expansion of autologous tumor-specific T cells and function as a unique TAA for this malignancy [49]. Importantly, SLAMF3 may serve as marker of bone marrow from MM patients and thus allowing identification of myeloma cells by flow cytometry and immunohistochemistry. Silencing of SLAMF3 in MM cells decreased the number of viable myeloma cells and enhanced the anti-tumor activity of conventional chemotherapeutics. These findings suggest that SLAMF3 can be considered as a therapeutic target to induce complement and cell-mediated lysis of myeloma cells [50]. These suggestions were further reinforced by Atanackovic et al. who showed that SLAMF3 is the only over-expressed/phosphorylated immunoreceptor in myeloma cell lines and primary CD138- negative cell population, which have previously been recognized as myeloma precursors cells [50] (Table 1). High expression of SLAMF3 has been described in patients with monoclonal gammopathies of uncertain significance, smoldering myeloma, and leukemia. The SLAMF3-targeting strategy was extended successfully to plasma cells from patients with MGUS [51]. Above all, the high expression of SLAMF3 correlates well with chemotherapy-resistant cells and myeloma-propagating phenotype. Thus, designing monoclonal antibodies specific to SLAMF3 could improve the treatment and help to obtain prolonged remissions in MM patients.

### SLAMF4

SLAMF4 (CD244, 2B4) expression is reported on CD8+ T cells, γδ T cells, NK cells, macrophages, basophils, mast cells and eosinophils. Following interaction of SLAMF4 with its natural ligand, SLAMF2, its cytoplasmic domain binds to SAP [21] (Figure 1). This interaction activates NK cell and induces IFN-γ secretion [52]. SLAMF4-mediated activation of NK cells involves complex interactions involving LAT, Ras, Raf, ERK and p38 [53]. In SLAMF4-deficient mice, NK cells are ineffective in eliminating cancer cells because they are inactivated. Functional impairment of NK cells in the absence of SLAMF4/SLAMF2 interactions is associated with defective calcium signaling as well [54].

In acute myeloid leukemia patients, polyclonal large granular lymphocytes (LGLs) were reported to have anti-tumor activity. The LGLs are CD3^+^/CD8^+^/CD56^+^, polyclonal cells that do not express NK cell receptors for MHC class I molecules. SLAMF4 expressed by LGLs largely contributes to their ability to lyse leukemia cells of patients [55]. SLAMF4 might potentiate the activator signal through T lymphocyte receptor (TCR) ζ chain. Indeed, the chimeric SLAMF4-TCRζ receptor induces more specific cytolysis of leukemia cells than SLAMF4 alone. This observation has also been extended to memory T cells stimulated by the tumor antigen [56]. SLAMF4 receptor has potent stimulatory effect in NK cells when expressed alone or as an antigen-specific SLAMF4- TCRζ chimeric receptor. Thus, SLAMF4 could be considered as powerful new tool for adoptive immunotherapy of leukemia and other malignancies [57] (Table 2).

### SLAMF5

SLAMF5 (CD84) expression has been described in thymocytes (highest by single positive cells), T cells, T_FH_ cells, B cells (overexpressed with activation), macrophages, DCs, platelets, basophils, mast cells and eosinophils. SLAMF5 is involved in T cells activation by enhancing IFN-γ secretion [58] and in the stimulation of platelets [21, 59]. SLAMF5 could also serve to distinguish two splenic B cells populations: SLAMF5^Low^ and SLAMF5^high^. SLAMF5^high^ sub-population represents a subset of memory B cells as demonstrated by increased cell size, co-expression of the memory B cell-specific marker CD27, somatically mutated Ig variable region genes, and increased proliferation compared to SLAMF5^Low^ B cells. The ligation of SLAMF5 with a specific mAb induces rapid phosphorylation of tyrosine residues in ITSM motif. Then, SLAMF5 recruits the cytoplasmic adaptor proteins SAP and EAT-2 [60] (Figure 1).

Among the CLL characteristics are the accumulation of CD5+ B lymphocytes in peripheral blood, in lymphoid organs and in bone marrow and the accumulation of the malignant cells due to reduced apoptosis sensitivity. CLL may be classified based on mutational status of the immunoglobulin variable heavy-chain gene, ZAP-70 overexpression, cytogenetic abnormalities (13q-, + 12, 11q-, 17p-) and expression of several cell surface antigens (CD38, CD49d) that correlate with risk of disease progression (Rai or Binet staging system) [61]. In the context of CLL, SLAMF5 expression is significantly higher during early stages of disease and is regulated by macrophage migration inhibitory factor and its receptor CD74 (Table 1). The activation of SLAMF5 initiates Akt signaling cascade that increases the anti-apoptotic Bcl-2 gene expression and consequently enhances CLL cell survival. Thus, the blockade of the SLAMF5-dependent survival pathway in CLL cells might represent a new therapeutic strategy [62]. Indeed, the blockade of SLAMF5 inhibits the interaction between CLL cells and their microenvironment inducing cell death as a consequence [63]. Several studies have reported a panel of clusters of differentiation (CD) antigens that are known to correlate with the prognosis of CLL. Huang et al. identified the profiles of surface CD antigens that distinguish clinically stable from progressive or slow progressive CLL. In the case of progressive CLL, 27 CD antigens including SLAMF5, are differentially abundant (CD11a, CD11b, CD11c, CD18, CD19, CD20, CD21, CD22, CD23, CD24, CD25, CD38, CD40, CD43, CD45, CD45RA, CD52, CD69, CD81, CD98, CD102, CD148, CD180, CD196 and CD270). This profiling provided the basis for a rapid test to identify patients with CLL according to the probability of clinical progression and the potential of earlier requirement for treatment [61].

### SLAMF6

SLAMF6 (CD352, NTB-A, SF2000, Ly108 in mice) expression is reported in thymocytes (highest by double negative and single positive cells), T cells, B cells (overexpressed with activation), DCs, NK cells, neutrophils, eosinophils. It is also expressed under two distinct isoforms (LY108–1 and LY108–2) in lupus-prone mice [21]. In NK cells, SLAMF6 activation is implicated in the cytotoxic activities and cytokine production [64]. SLAMF6 plays a key role in T cell activation [65] and neutrophil functions [66]. In pathological conditions, SLAMF6 is highly expressed in CLL and B lymphocytes of lymphoma patients (Table 1). For this reason, Korver et al., used two mAbs (994.1 and 480.12) to target SLAMF6 in a preclinical study of CLL and lymphoma, (Table 3). Indeed, they showed 86% decrease of tumor volume in a xenograft nude mouse CA46 model treated with 300μg of mAbs twice a week. Moreover, in a xenograft SCID mouse and in systemically disseminated Raji human lymphoma cells model, authors observed significant increase in survival of animals injected with 100 μg of mAbs [67]. These promising findings highlight the importance of targeting SLAMF6 as an immunotherapy for B-cell malignancies.

The decrease in CLL progression observed by Yigit in xenografted SCID mice model after the administration of anti-SLAMF6 confirmed the implication of this receptor in CLL pathophysiology. The effect of anti-SLAMF6 is amplified when it was administered concomitantly with Ibrutinib, a Bruton tyrosine kinase inhibitor. Anti-SLAMF6 decreased the number of CLL cells everywhere except in peritoneal cavity and Ibrutinib increased the efficiency of anti-SLAMF6 by inducing the release of CLL cells from their niche. All these data suggest that the combination of anti-SLAMF6 and Ibrutinib might be an efficient new treatment against CLL [68].

### SLAMF7

**Figure 3:**
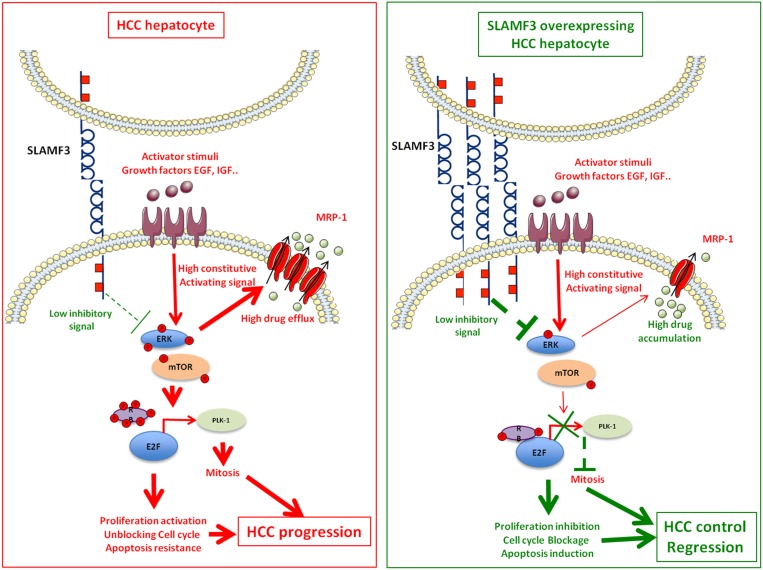
SLAMF3 implication model as hepatocellular carcinoma (HCC) Left quadrant, shows the schematic representation of the role of SLAMF3 as negative regulator of MAPK / mTOR pathway. In HCC hepatocyte, the rate of SLAMF3 is lowered. In this cell, the triggered signals by growth factors through their specific receptors activate signalization pathways, including the MAPK pathway, which will be constitutively active. This pathway stimulates, among others, the expression of certain resistance transporters such as MRP-1 that are responsible for the resistance to chemotherapies molecules. The MAPK pathway also modulates the formation of the nuclear complex RB / E2F, whose target genes are among that regulating the cell cycle and mitosis such as Plk-1. The resultant of these pathways activation state are enhanced cell proliferation, unblocked cell cycle and apoptosis escape leading to the progression of the tumor mass. Right quadrant, represents the hypothetic therapeutic model of SLAMF3 in HCC. The restoration of a high expression of SLAMF3 induced a down phosphorylation of MAPK ERK and mTOR. First, the strong expression of SLAMF3 reduced phosphorylation ratio of RB permitting its binding to the E2F factor which induces its inhibition. The decreased Plk-1 expression, one important E2F target is implicated in the inhibition of mitosis induced by the high expression of SLAMF3. Second, the high expression of SLAMF3 inhibits expression and function of MRP-1, that sensitize the cancer cell to drugs. Finally, the high expression of SLAMF3 in the cancer cell controls proliferation, blocks mitosis and sensitizes to apoptosis which controls progression of the tumor mass.

NK cells, T lymphocytes, activated B cells and macrophages are the cells that majorly express SLAMF7 (CD319, CS1, CRACC) [21] (Figure 1). The cytoplasmic domain of SLAMF7 contains two ITSM that are involved in the interaction with SAP. The SLAMF7 homophilic interaction regulates NK cell cytolytic activity [69]. SLAMF7 is highly expressed by more than 97% of myeloma cells while its expression is restricted in normal cells [70, 71]. In some preclinical studies, SLAMF7 was targeted by humanized mAb, HuLuc63 (Elotuzumab), which stained CD138+ myeloma cells, NK cells, NK-like T cells, and CD8+ T cells [70] (Table 2). HuLuc63 inhibits MM cells binding to bone marrow stromal cells and induces antibody-dependent cellular cytotoxicity against MM cells (Figure 3). This effect is SLAMF7-specific and dose-dependent, and leads to tumor regression in multiple xenograft models of human MM [70, 72]. Moreover, Elotuzumab, promoted the ligation between NK and myeloma cells by the intermediary of SLAMF7 and induced NK cytotoxicity against myeloma cells independent of ADCC [73, 74]. In addition, SLAMF7-derived peptides showed efficacy to induce the activation of some cytotoxic T cells clones. Later, Bae et al. suggested SLAMF7_239–247_ as SLAMF7-specific HLA-A2 peptide capable of activating cytotoxic T lymphocytes and inducing specifically the lysis of the primary MM cells and HLA-A2^+^-CS1^+^ MM cell line [75] (Table 3).

Several studies showed that Elotuzumab could trigger autologous ADCC against primary MM cells resistant to conventional or novel therapies like Bortezomib and HSP90 inhibitors. Pretreatment with conventional or novel anti-MM drugs enhance HuLuc63-induced MM cell lysis [71] (Figure 2). Therefore, these results prove that SLAMF7 play a pivotal role in the elimination of MM and provide the preclinical rationale to test it alone or in combination with other drugs in clinical trials. Bortezomib is a reversible proteasome inhibitor with significant activity in MM that could enhance Elotuzumab activity. When tested in MM xenograft model, the combination of Elotuzumab and Bortezomib, exhibited a significant enhancement of *in vivo* antitumor activity [71]. These preclinical results encouraged phase I and II clinical trials of Elotuzumab in patients with relapsed or refractory MM. When tested in phase I, Elotuzumab alone failed to reproduce the same efficiency as in animal model [76]. On the other hand, when administered to patients under Bortezomib treatment, Elotuzumab improved the objective response rate from 45% to 48% [77]. In a phase II trial, the combined use of Elotuzumab with Lenalidomide and dexamethasone in 150 patients with refractory/relapsed MM, seems to increase the progression-free survival from 6.9 to 9.7 months with stable ORR from 66% to 63% [78]. Importantly, a positive effect of Elotuzumab is reported to improve the ORR to 82% when used in combination with Lenalidomide and low-dose of dexamethasone [79].

In a phase II, randomized, multicenter, open-label, intravenous Elotuzumab (10 or 20 mg daily) in combination with oral Lenalidomide (25 mg daily) and dexamethasone (40 mg daily) improves the efficacy with an acceptable safety in patients with relapsed MM. These results seem to be better when only Lenalidomide and dexamethasone are used [80]. In patients with renal impairment, (about 50% of patients with MM), Elotuzumab might be used without dose adjustment but Lenalidomide needed a dose adjustment [81]. All these findings allowed the realization of a phase III testing of Elotuzumab in combination with Lenalidomide and dexamethasone in relapsed MM and in untreated MM patients [82]. In patients with relapsed or refractory MM, combination of Elotuzumab, Lenalidomide, and dexamethasone (Elotuzumab group: 325 patients) significantly reduced the risk of disease progression or death by 30% compared to control (control group: 321 patients treated with Lenalidomide and Dexamethasone). However, this study did not integrate Lenalidomide untreated patients as control group. This lack of control makes it difficult to conclude about the real effect of adding Lenalidomide. It has to be noted that the adverse events reported in 25% of the patients were principally common hematologic toxic effects (lymphocytopenia, anemia, thrombocytopenia and neutropenia). Serious adverse events were reported in 65% and 57% of patients in the Elotuzumab and the control group, respectively. In the Elotuzumab group, 34% patients had grade 3 or 4 neutropenia, as compared with 44% in the control group. Grade 3 or 4 lymphocytopenia was reported in 77% patients in the Elotuzumab group and 49% in the control group. Other reported side effects were non-hematologic toxic effects like fatigue, diarrhea and muscle spasms [82]. The above-mentioned findings allowed the FDA, in November 2015, to issue the authorization of Elotuzumab use in combination with Lenalidomide and dexamethasone in MM.

Lately, the combination of Elotuzumab with Thalidomide and dexamethasone was tested in a phase II trial involving 40 patients with refractory/relapsed MM. The results show an ORR of 38% with median progression-free survival of 3.9 months. This combination might constitute a potential alternative to the Elotuzumab, Lenalidomide and dexamethasone combination [83].

### SLAM in solid cancers

Other cell types outside the immune system also express SLAM members. The reported detection of SLAMF2 in melanoma and SLAMF3 in hepatocellular carcinoma (HCC) assigned new roles to SLAM receptors in physiology and pathology. These recent observations reveal numerous potential therapeutic applications targeting SLAM members in solid cancers (Table 3).

### SLAMF1

Quite often, SLAMF1 is expressed on normal and malignant cells of the immune system [84]. However, little is known about its expression outside the hematopoietic system. Although SLAMF1 is not found in different regions of normal brain tissues, immunohistochemical study revealed its expression in 77.6% of human central nervous system (CNS) tumors, including glioblastoma, anaplastic astrocytoma, diffuse astrocytoma and ependymoma. These observations have proposed the expression of new isoform of SLAMF1 nCD150 as new diagnostic marker in CNS tumors [85] (Table 2).

Mehrle et al. reported that the up-regulation or silencing of SLAMF1 in human lymphocytes was accompanied by increased or decreased cytotoxic activity respectively, and suggested the implication of SLAMF1 in cytotoxicity against human colon cancer. Moreover, increased cytotoxic activity against cancerous cells and IFN-secretion are the result of the up-regulation of SLAMF1 in T cells that decrease the tumor progression index in xenografted mice [86]. Following its activation, SLAMF1 co-localizes to the lipid rafts (specific membrane compartments) in cytotoxic CD8+ T cells.

### SLAMF2/SLAMF4

NK cells play a crucial role in the anti-cancer immunity. In patients with advanced-stage HCC, Attenuated infiltration and dysfunction of NK cells in the intratumoral region was positively associated with the increased level of activated monocytes/macrophages in peritumoral stroma of HCC tissues. Accordingly, activated monocytes isolated from HCC tissues caused transient activation and consequently their apoptosis. This process was mediated by cell-cell interactions by way of SLAMF2-SLAMF4, but not NKG2D and NKp30 [87].

In Melanoma, targeting the interaction of SLAMF2-SLAMF4 or use of anti-SLAMF4 mAb are promising therapeutic strategies. The activation of SLAMF4 or SLAMF2 in murine tumor model by injecting specific antibodies resulted in a five-fold reduction of melanoma cells metastasis. This anti-tumor effect involves cytolytic function and IFN-γ production of NK cells as expected [88].

The implication of SLAMF2-SLAMF4 in melanoma anti-tumor effect is subject of some controversies. For instance, in one study involving SLAMF4 (−/−) mice, it was difficult to confirm the role of SLAMF2-SLAMF4 interaction in anti-melanoma response. When injected with SLAMF2+ and SLAMF2- metastatic B16 melanoma cells, wild-type mice poorly rejected the SLAMF2+ melanoma cells compared to SLAMF2- tumor cells, suggesting an inhibitory effect of SLAMF4-SLAMF2 interaction on melanoma cells [89].

### SLAMF3

Recently, it has been reported that SLAMF3 is the only member of its family expressed in hepatocytes [90] (Figure 1). Identifying SLAMF3 in non-blood tissue such as liver tissue opened new avenues to investigate other physiological functions of this receptor. SLAMF3 expression is high in human healthy primary hepatocytes and is reduced in HCC cells. SLAMF3 low expression was confirmed in several HCC cell lines suggesting its negative regulation in cancer cells [90] (Table 3). The restoration of SLAMF3 expression in these cells inhibited cell proliferation, migration and enhanced apoptosis (Figure 3). This finding suggested that SLAMF3 is involved in the control of hepatocyte proliferation and in carcinogenesis.

SLAMF3 controls proliferation in part by decreasing MAPK Erk1/2, JNK and mTOR phosphorylation [90]. SLAMF3 expression in HCC retains Retinoblastoma (RB) factor in its hypo-phosphorylated active form, which in turn inactivates E2F transcription factor, thereby repressing the expression and activation of Polo-like kinase Plk-1 (Figure 3). A clear inverse correlation was also observed between SLAMF3 and Plk-1 expression in patients with HCC [91]. Thus, the inhibitory effect of SLAMF3 on cell cycle progression occurs through Plk-1 and RB activation [91]. These findings attributed new functions to SLAMF3 as molecular regulator of cell proliferation, cell cycle progression and apoptosis and qualify SLAMF3 to be considered as potential therapeutic candidate to control tumor progression at least in the liver.

In HCC, the expression of multi-drug resistance proteins, MRP-1, correlates with the resistance to chemotherapy and to treatment failure. MRP-1 (ABCC1), a member of the ABC superfamily transporters is expressed in all organs. MRP-1 mediates active efflux of a broad range of physiological substrates like glucuronide, glutathione and sulfate conjugates [92]. Chemotherapy drugs including Doxorubicin, Vincristine, Etoposide and Methotrexate also induce these reagents [93]. In HCC cells, induction of SLAMF3 expression decreased the expression of MRP-1. Furthermore, restoring high SLAMF3 expression *in vitro* model inhibited the efflux of therapeutic molecules [94] (Figure 3). Elevated SLAMF3 expression might thus play a key role in eliminating cancer cells by the chemotherapy drugs [95].

## CONCLUSIONS AND PERSPECTIVES

SLAM molecules are of particular interest for diagnosis and therapy of various cancers. Targeting these molecules is already under clinical investigations and has the potential to open new therapeutic options to improve management of cancer patients (Table 2). The most advanced therapeutic strategy is targeting of SLAMF7 (CS1) using humanized mAb in MM management. Completion of phase III studies and validation of therapeutic efficacy and safety of Elotuzumab in MM requires addressing of key issues regarding the biology of SLAMF7.

Another key finding is the SLAMF3 expression in hepatic tissue and its role as cell proliferation regulator and apoptosis inductor. This regulation involves several pathways constitutively activated in HCC such as MAPK pathways. However, comprehensive investigations are needed to identify SLAMF3 molecular partners to clarify the link between receptor expression and regression of tumors in xenografts Nude mice model. The implication of immune cells (innate and adaptive) cannot be ignored and requires further investigation. In parallel, various risk factors that promote transformation of cells like viral factors, metabolic abnormalities or the intake of alcohol could have an impact on the SLAM receptor expression or function. Further work is needed to determine the cross talk between these risk factors and SLAM.

## References

[1] Cannons JL, Tangye SG, Schwartzberg PL (2011). SLAM family receptors and SAP adaptors in immunity. Annu Rev Immunol.

[2] Detre C, Keszei M, Romero X, Tsokos GC, Terhorst C (2010). SLAM family receptors and the SLAM-associated protein (SAP) modulate T cell functions. Semin Immunopathol.

[3] Chen R, Latour S, Shi X, Veillette A (2006). Association between SAP and FynT: inducible SH3 domain-mediated interaction controlled by engagement of the SLAM receptor. Mol Cell Biol.

[4] Sayos J, Wu C, Morra M, Wang N, Zhang X, Allen D, van Schaik S, Notarangelo L, Geha R, Roncarolo MG, Oettgen H, De Vries JE, Aversa G, Terhorst C (1998). The X-linked lymphoproliferative-disease gene product SAP regulates signals induced through the co-receptor SLAM. Nature.

[5] Shlapatska LM, Mikhalap SV, Berdova AG, Zelensky OM, Yun TJ, Nichols KE, Clark EA, Sidorenko SP (2001). CD150 association with either the SH2-containing inositol phosphatase or the SH2-containing protein tyrosine phosphatase is regulated by the adaptor protein SH2D1A. J Immunol.

[6] Calpe S, Wang N, Romero X, Berger SB, Lanyi A, Engel P, Terhorst C (2008). The SLAM and SAP Gene Families Control Innate and Adaptive Immune Responses. Advances in Immunology. Elsevier.

[7] Fennelly JA, Tiwari B, Davis SJ, Evans EJ (2001). CD2F-10: a new member of the CD2 subset of the immunoglobulin superfamily. Immunogenetics.

[8] Kingsbury GA, Feeney LA, Nong Y, Calandra SA, Murphy CJ, Corcoran JM, Wang Y, Prabhu Das MR, Busfield SJ, Fraser CC, Villeval JL (2001). Cloning, expression, and function of BLAME, a novel member of the CD2 family. J Immunol.

[9] Sandrin MS, Gumley TP, Henning MM, Vaughan HA, Gonez LJ, Trapani JA, McKenzie IF (1992). Isolation and characterization of cDNA clones for mouse Ly-9. J Immunol.

[10] Sandrin MS, Henning MM, Lo MF, Baker E, Sutherland GR, McKenzie IF (1996). Isolation and characterization of cDNA clones for Humly9: the human homologue of mouse Ly9. Immunogenetics.

[11] Ostrakhovitch EA, Li SS (2006). The role of SLAM family receptors in immune cell signaling. Biochem Cell Biol.

[12] Morra M, Howie D, Grande MS, Sayos J, Wang N, Wu C, Engel P, Terhorst C (2001). X-linked lymphoproliferative disease: a progressive immunodeficiency. Annu Rev Immunol.

[13] Roncagalli R, Taylor JE, Zhang S, Shi X, Chen R, Cruz-Munoz ME, Yin L, Latour S, Veillette A (2005). Negative regulation of natural killer cell function by EAT-2, a SAP-related adaptor. Nat Immunol.

[14] Veillette A (2006). NK cell regulation by SLAM family receptors and SAP-related adapters. Immunol Rev.

[15] Simarro M, Lanyi A, Howie D, Poy F, Bruggeman J, Choi M, Sumegi J, Eck MJ, Terhorst C (2004). SAP increases FynT kinase activity and is required for phosphorylation of SLAM and Ly9. Int Immunol.

[16] Del Valle JM, Engel P, Martín M (2003). The cell surface expression of SAP-binding receptor CD229 is regulated via its interaction with clathrin-associated adaptor complex 2 (AP-2). J Biol Chem.

[17] Schlessinger J (2000). Cell signaling by receptor tyrosine kinases. Cell.

[18] Martín M, Del Valle JM, Saborit I, Engel P (2005). Identification of Grb2 as a novel binding partner of the signaling lymphocytic activation molecule-associated protein binding receptor CD229. J Immunol.

[19] Bouchon A, Cella M, Grierson HL, Cohen JI, Colonna M (2001). Activation of NK cell-mediated cytotoxicity by a SAP-independent receptor of the CD2 family. J Immunol.

[20] Henning G, Kraft MS, Derfuss T, Pirzer R, de Saint-Basile G, Aversa G, Fleckenstein B, Meinl E (2001). Signaling lymphocytic activation molecule (SLAM) regulates T cellular cytotoxicity. Eur J Immunol.

[21] Schwartzberg PL, Mueller KL, Qi H, Cannons JL (2009). SLAM receptors and SAP influence lymphocyte interactions, development and function. Nat Rev Immunol.

[22] Engel P, Eck MJ, Terhorst C (2003). The SAP and SLAM families in immune responses and X-linked lymphoproliferative disease. Nat Rev Immunol.

[23] Muccio VE, Saraci E, Gilestro M, Gattei V, Zucchetto A, Astolfi M, Ruggeri M, Marzanati E, Passera R, Palumbo A, Boccadoro M, Omedè P (2016). Multiple myeloma: new surface antigens for the characterization of plasma cells in the era of novel agents. Cytometry B Clin Cytom.

[24] Bologna C, Buonincontri R, Serra S, Vaisitti T, Audrito V, Brusa D, Pagnani A, Coscia M, D’Arena G, Mereu E, Piva R, Furman RR, Rossi D (2016). SLAMF1 regulation of chemotaxis and autophagy determines CLL patient response. J Clin Invest.

[25] Schweighofer CD, Coombes KR, Barron LL, Diao L, Newman RJ, Ferrajoli A, O’Brien S, Wierda WG, Luthra R, Medeiros LJ, Keating MJ, Abruzzo LV (2011). A Two-Gene Signature, SKI and SLAMF1, Predicts Time-to-Treatment in Previously Untreated Patients with Chronic Lymphocytic Leukemia. PLoS ONE.

[26] Tatsuo H, Ono N, Tanaka K, Yanagi Y (2000). SLAM (CDw150) is a cellular receptor for measles virus. Nature.

[27] Lühl NC, Zirngibl F, Dorneburg C, Wei J, Dahlhaus M, Barth TF, Meyer LH, Queudeville M, Eckhoff S, Debatin KM, Beltinger C (2014). Attenuated measles virus controls pediatric acute B-lineage lymphoblastic leukemia in NOD/SCID mice. Haematologica.

[28] Takeda S, Kanbayashi D, Kurata T, Yoshiyama H, Komano J (2014). Enhanced susceptibility of B lymphoma cells to measles virus by Epstein-Barr virus type III latency that upregulates CD150/signaling lymphocytic activation molecule. Cancer Sci.

[29] Miest TS, Frenzke M, Cattaneo R (2013). Measles virus entry through the signaling lymphocyte activation molecule governs efficacy of mantle cell lymphoma radiovirotherapy. Mol Ther.

[30] Yurchenko MY, Kovalevska LM, Shlapatska LM, Berdova GG, Clark EA, Sidorenko SP (2010). CD150 regulates JNK1/2 activation in normal and Hodgkin’s lymphoma B cells. Immunol Cell Biol.

[31] Yurchenko MY, Kashuba EV, Shlapatska LM, Sivkovich SA, Sidorenko SP (2005). The role of CD150-SH2D1A association in CD150 signaling in Hodgkin’s lymphoma cell lines. Exp Oncol.

[32] Yurchenko M, Shlapatska LM, Romanets OL, Ganshevskiy D, Kashuba E, Zamoshnikova A, Ushenin YV, Snopok BA, Sidorenko SP (2011). CD150-mediated Akt signalling pathway in normal and malignant B cells. Exp Oncol.

[33] Kato K, Koyanagi M, Okada H, Takanashi T, Wong YW, Williams AF, Okumura K, Yagita H (1992). CD48 is a counter-receptor for mouse CD2 and is involved in T cell activation. J Exp Med.

[34] Sandrin MS, Mouhtouris E, Vaughan HA, Warren HS, Parish CR (1993). CD48 is a low affinity ligand for human CD2. J Immunol.

[35] Greenaway S, Henniker AJ, Walsh M, Bradstock KF (1994). A pilot clinical trial of two murine monoclonal antibodies fixing human complement in patients with chronic lymphatic leukaemia. Leuk Lymphoma.

[36] Sun H, Norris BJ, Atkinson K, Biggs JC, Smith GM (1998). Preclinical antitumor activity of an antibody against the leukocyte antigen CD48. Clin Cancer Res.

[37] Hosen N, Ichihara H, Mugitani A, Aoyama Y, Fukuda Y, Kishida S, Matsuoka Y, Nakajima H, Kawakami M, Yamagami T, Fuji S, Tamaki H, Nakao T (2012). CD48 as a novel molecular target for antibody therapy in multiple myeloma. Br J Haematol.

[38] Boles NC, Lin KK, Lukov GL, Bowman TV, Baldridge MT, Goodell MA (2011). CD48 on hematopoietic progenitors regulates stem cells and suppresses tumor formation. Blood.

[39] Vaughan HA, Thompson CH, Sparrow RL, McKenzie IF (1983). Hu Ly-M3—a human leukocyte antigen. Transplantation.

[40] Romero X, Zapater N, Calvo M, Kalko SG, de la Fuente MA, Tovar V, Ockeloen C, Pizcueta P, Engel P (2005). CD229 (Ly9) lymphocyte cell surface receptor interacts homophilically through its N-terminal domain and relocalizes to the immunological synapse. J Immunol.

[41] Sintes J, Vidal-Laliena M, Romero X, Tovar V, Engel P (2007). Characterization of mouse CD229 (Ly9), a leukocyte cell surface molecule of the CD150 (SLAM) family. Tissue Antigens.

[42] Graham DB, Bell MP, McCausland MM, Huntoon CJ, van Deursen J, Faubion WA, Crotty S, McKean DJ (2006). Ly9 (CD229)-deficient mice exhibit T cell defects yet do not share several phenotypic characteristics associated with SLAM- and SAP-deficient mice. J Immunol.

[43] Chatterjee M, Rauen T, Kis-Toth K, Kyttaris VC, Hedrich CM, Terhorst C, Tsokos GC (2012). Increased expression of SLAM receptors SLAMF3 and SLAMF6 in systemic lupus erythematosus T lymphocytes promotes Th17 differentiation. J Immunol.

[44] Rosenberg SA (1997). Cancer vaccines based on the identification of genes encoding cancer regression antigens. Immunol Today.

[45] Van den Eynde BJ, van der Bruggen P (1997). T cell defined tumor antigens. Curr Opin Immunol.

[46] Van Pel A, van der Bruggen P, Coulie PG, Brichard VG, Lethé B, van den Eynde B, Uyttenhove C, Renauld JC, Boon T (1995). Genes coding for tumor antigens recognized by cytolytic T lymphocytes. Immunol Rev.

[47] Hansen OP, Jessen B, Videbaek A (1973). Prognosis of myelomatosis on treatment with prednisone and cytostatics. Scand J Haematol.

[48] de la Fuente J, Merx K, Steer EJ, Müller M, Szydlo RM, Maywald O, Berger U, Hehlmann R, Goldman JM, Cross NC, Melo JV, Hochhaus A, German CML Study Group (2001). ABL-BCR expression does not correlate with deletions on the derivative chromosome 9 or survival in chronic myeloid leukemia. Blood.

[49] Bund D, Mayr C, Kofler DM, Hallek M, Wendtner CM (2006). Human Ly9 (CD229) as novel tumor-associated antigen (TAA) in chronic lymphocytic leukemia (B-CLL) recognized by autologous CD8+ T cells. Exp Hematol.

[50] Atanackovic D, Panse J, Hildebrandt Y, Jadczak A, Kobold S, Cao Y, Templin J, Meyer S, Reinhard H, Bartels K, Lajmi N, Zander AR, Marx AH (2011). Surface molecule CD229 as a novel target for the diagnosis and treatment of multiple myeloma. Haematologica.

[51] Carulli G, Buda G, Azzarà A, Ciancia EM, Sammuri P, Domenichini C, Guerri V, Petrini M (2016). CD229 Expression on Bone Marrow Plasma Cells from Patients with Multiple Myeloma and Monoclonal Gammopathies of Uncertain Significance. Acta Haematol.

[52] Tangye SG, Phillips JH, Lanier LL, Nichols KE (2000). Functional requirement for SAP in 2B4-mediated activation of human natural killer cells as revealed by the X-linked lymphoproliferative syndrome. J Immunol.

[53] Chuang SS, Kumaresan PR, Mathew PA (2001). 2B4 (CD244)-mediated activation of cytotoxicity and IFN-gamma release in human NK cells involves distinct pathways. J Immunol.

[54] Lee KM, Forman JP, McNerney ME, Stepp S, Kuppireddi S, Guzior D, Latchman YE, Sayegh MH, Yagita H, Park CK, Oh SB, Wülfing C, Schatzle J (2006). Requirement of homotypic NK-cell interactions through 2B4(CD244)/CD48 in the generation of NK effector functions. Blood.

[55] Costello RT, Sivori S, Mallet F, Sainty D, Arnoulet C, Reviron D, Gastaut JA, Moretta A, Olive D (2002). A novel mechanism of antitumor response involving the expansion of CD3+/CD56+ large granular lymphocytes triggered by a tumor-expressed activating ligand. Leukemia.

[56] Altvater B, Landmeier S, Pscherer S, Temme J, Juergens H, Pule M, Rossig C (2009). 2B4 (CD244) signaling via chimeric receptors costimulates tumor-antigen specific proliferation and *in vitro* expansion of human T cells. Cancer Immunol Immunother.

[57] Altvater B, Landmeier S, Pscherer S, Temme J, Schweer K, Kailayangiri S, Campana D, Juergens H, Pule M, Rossig C (2009). 2B4 (CD244) signaling by recombinant antigen-specific chimeric receptors costimulates natural killer cell activation to leukemia and neuroblastoma cells. Clin Cancer Res.

[58] Martin M, Romero X, de la Fuente MA, Tovar V, Zapater N, Esplugues E, Pizcueta P, Bosch J, Engel P (2001). CD84 functions as a homophilic adhesion molecule and enhances IFN-gamma secretion: adhesion is mediated by Ig-like domain 1. J Immunol.

[59] Nanda N, Andre P, Bao M, Clauser K, Deguzman F, Howie D, Conley PB, Terhorst C, Phillips DR (2005). Platelet aggregation induces platelet aggregate stability via SLAM family receptor signaling. Blood.

[60] Tangye SG, van de Weerdt BC, Avery DT, Hodgkin PD (2002). CD84 is up-regulated on a major population of human memory B cells and recruits the SH2 domain containing proteins SAP and EAT-2. Eur J Immunol.

[61] Huang PY, Best OG, Almazi JG, Belov L, Davis ZA, Majid A, Dyer MJ, Pascovici D, Mulligan SP, Christopherson RI (2014). Cell surface phenotype profiles distinguish stable and progressive chronic lymphocytic leukemia. Leuk Lymphoma.

[62] Binsky-Ehrenreich I, Marom A, Sobotta MC, Shvidel L, Berrebi A, Hazan-Halevy I, Kay S, Aloshin A, Sagi I, Goldenberg DM, Leng L, Bucala R, Herishanu Y (2014). CD84 is a survival receptor for CLL cells. Oncogene.

[63] Marom A, Barak AF, Kramer MP, Lewinsky H, Binsky-Ehrenreich I, Cohen S, Tsitsou-Kampeli A, Kalchenko V, Kuznetsov Y, Mirkin V, Dezorella N, Shapiro M, Schwartzberg PL (2017). CD84 mediates CLL-microenvironment interactions. Oncogene.

[64] Flaig RM, Stark S, Watzl C (2004). Cutting edge: NTB-A activates NK cells via homophilic interaction. J Immunol.

[65] Valdez PA, Wang H, Seshasayee D, van Lookeren Campagne M, Gurney A, Lee WP, Grewal IS (2004). NTB-A, a new activating receptor in T cells that regulates autoimmune disease. J Biol Chem.

[66] Howie D, Laroux FS, Morra M, Satoskar AR, Rosas LE, Faubion WA, Julien A, Rietdijk S, Coyle AJ, Fraser C, Terhorst C (2005). Cutting edge: the SLAM family receptor Ly108 controls T cell and neutrophil functions. J Immunol.

[67] Korver W, Singh S, Liu S, Zhao X, Yonkovich S, Sweeney A, Anton K, Lomas WE, Greenwood R, Smith A, Tran DH, Shinkawa P, Jimenez M (2007). The lymphoid cell surface receptor NTB-A: a novel monoclonal antibody target for leukaemia and lymphoma therapeutics. Br J Haematol.

[68] Yigit B, Halibozek PJ, Chen SS, O’Keeffe MS, Arnason J, Avigan D, Gattei V, Bhan A, Cen O, Longnecker R, Chiorazzi N, Wang N, Engel P, Terhorst C (2016). A combination of an anti-SLAMF6 antibody and ibrutinib efficiently abrogates expansion of chronic lymphocytic leukemia cells. Oncotarget.

[69] Kumaresan PR, Lai WC, Chuang SS, Bennett M, Mathew PA (2002). CS1, a novel member of the CD2 family, is homophilic and regulates NK cell function. Mol Immunol.

[70] Hsi ED, Steinle R, Balasa B, Szmania S, Draksharapu A, Shum BP, Huseni M, Powers D, Nanisetti A, Zhang Y, Rice AG, van Abbema A, Wong M (2008). CS1, a potential new therapeutic antibody target for the treatment of multiple myeloma. Clin Cancer Res.

[71] van Rhee F, Szmania SM, Dillon M, van Abbema AM, Li X, Stone MK, Garg TK, Shi J, Moreno-Bost AM, Yun R, Balasa B, Ganguly B, Chao D (2009). Combinatorial efficacy of anti-CS1 monoclonal antibody elotuzumab (HuLuc63) and bortezomib against multiple myeloma. Mol Cancer Ther.

[72] Tai YT, Dillon M, Song W, Leiba M, Li XF, Burger P, Lee AI, Podar K, Hideshima T, Rice AG, van Abbema A, Jesaitis L, Caras I (2008). Anti-CS1 humanized monoclonal antibody HuLuc63 inhibits myeloma cell adhesion and induces antibody-dependent cellular cytotoxicity in the bone marrow milieu. Blood.

[73] Collins SM, Bakan CE, Swartzel GD, Hofmeister CC, Efebera YA, Kwon H, Starling GC, Ciarlariello D, Bhaskar S, Briercheck EL, Hughes T, Yu J, Rice A, Benson DM (2013). Elotuzumab directly enhances NK cell cytotoxicity against myeloma via CS1 ligation: evidence for augmented NK cell function complementing ADCC. Cancer Immunol Immunother.

[74] Guo H, Cruz-Munoz ME, Wu N, Robbins M, Veillette A (2015). Immune cell inhibition by SLAMF7 is mediated by a mechanism requiring src kinases, CD45, and SHIP-1 that is defective in multiple myeloma cells. Mol Cell Biol.

[75] Bae J, Song W, Smith R, Daley J, Tai YT, Anderson KC, Munshi NC (2012). A novel immunogenic CS1-specific peptide inducing antigen-specific cytotoxic T lymphocytes targeting multiple myeloma. Br J Haematol.

[76] Zonder JA, Mohrbacher AF, Singhal S, van Rhee F, Bensinger WI, Ding H, Fry J, Afar DE, Singhal AK (2012). A phase 1, multicenter, open-label, dose escalation study of elotuzumab in patients with advanced multiple myeloma. Blood.

[77] Jakubowiak AJ, Benson DM, Bensinger W, Siegel DS, Zimmerman TM, Mohrbacher A, Richardson PG, Afar DE, Singhal AK, Anderson KC, Phase I (2012). Phase I trial of anti-CS1 monoclonal antibody elotuzumab in combination with bortezomib in the treatment of relapsed/refractory multiple myeloma. J Clin Oncol.

[78] Jakubowiak A, Offidani M, Pégourie B, De La Rubia J, Garderet L, Laribi K, Bosi A, Marasca R, Laubach J, Mohrbacher A, Carella AM, Singhal AK, Tsao LC (2016). Randomized phase 2 study: elotuzumab plus bortezomib/dexamethasone vs bortezomib/dexamethasone for relapsed/refractory MM. Blood.

[79] Lonial S, Vij R, Harousseau JL, Facon T, Moreau P, Mazumder A, Kaufman JL, Leleu X, Tsao LC, Westland C, Singhal AK, Jagannath S (2012). Elotuzumab in combination with lenalidomide and low-dose dexamethasone in relapsed or refractory multiple myeloma. J Clin Oncol.

[80] Richardson PG, Jagannath S, Moreau P, Jakubowiak AJ, Raab MS, Facon T, Vij R, White D, Reece DE, Benboubker L, Zonder J, Tsao LC, Anderson KC, 1703 study investigators (2015). Elotuzumab in combination with lenalidomide and dexamethasone in patients with relapsed multiple myeloma: final phase 2 results from the randomised, open-label, phase 1b-2 dose-escalation study. Lancet Haematol.

[81] Berdeja J, Jagannath S, Zonder J, Badros A, Kaufman JL, Manges R, Gupta M, Tendolkar A, Lynch M, Bleickardt E, Paliwal P, Vij R (2016). Pharmacokinetics and Safety of Elotuzumab Combined With Lenalidomide and Dexamethasone in Patients With Multiple Myeloma and Various Levels of Renal Impairment: Results of a Phase Ib Study. Clin Lymphoma Myeloma Leuk.

[82] Lonial S, Dimopoulos M, Palumbo A, White D, Grosicki S, Spicka I, Walter-Croneck A, Moreau P, Mateos MV, Magen H, Belch A, Reece D, Beksac M, ELOQUENT-2 Investigators (2015). Elotuzumab Therapy for Relapsed or Refractory Multiple Myeloma. N Engl J Med.

[83] Mateos MV, Granell M, Oriol A, Martinez-Lopez J, Blade J, Hernandez MT, Martín J, Gironella M, Lynch M, Bleickardt E, Paliwal P, Singhal A, San-Miguel J (2016). Elotuzumab in combination with thalidomide and low-dose dexamethasone: a phase 2 single-arm safety study in patients with relapsed/refractory multiple myeloma. Br J Haematol.

[84] Veillette A (2006). Immune regulation by SLAM family receptors and SAP-related adaptors. Nat Rev Immunol.

[85] Romanets-Korbut O, Najakshin AM, Yurchenko M, Malysheva TA, Kovalevska L, Shlapatska LM, Zozulya YA, Taranin AV, Horvat B, Sidorenko SP (2015). Expression of CD150 in Tumors of the Central Nervous System: Identification of a Novel Isoform. PLOS ONE.

[86] Mehrle S, Schmidt J, Büchler MW, Watzl C, Märten A (2008). Enhancement of anti-tumor activity *in vitro* and *in vivo* by CD150 and SAP. Mol Immunol.

[87] Wu Y, Kuang DM, Pan WD, Wan YL, Lao XM, Wang D, Li XF, Zheng L (2013). Monocyte/macrophage-elicited natural killer cell dysfunction in hepatocellular carcinoma is mediated by CD48/2B4 interactions. Hepatology.

[88] Johnson LA, Vaidya SV, Goldfarb RH, Mathew PA (2003). 2B4(CD244)-mediated activation of NK cells reduces metastases of B16F10 melanoma in mice. Anticancer Res.

[89] Vaidya SV, Stepp SE, McNerney ME, Lee JK, Bennett M, Lee KM, Stewart CL, Kumar V, Mathew PA (2005). Targeted disruption of the 2B4 gene in mice reveals an *in vivo* role of 2B4 (CD244) in the rejection of B16 melanoma cells. J Immunol.

[90] Marcq I, Nyga R, Cartier F, Amrathlal RS, Ossart C, Ouled-Haddou H, Ghamlouch H, Galmiche A, Chatelain D, Lamotte L, Debuysscher V, Fuentes V, Nguyen-Khac E (2013). Identification of SLAMF3 (CD229) as an Inhibitor of Hepatocellular Carcinoma Cell Proliferation and Tumour Progression. PLoS ONE.

[91] Bouhlal H, Ouled-Haddou H, Debuysscher V, Singh AR, Ossart C, Reignier A, Hocini H, Fouquet G, Al Baghami M, Eugenio MS, Nguyen-Khac E, Regimbeau JM, Marcq I (2016). RB/PLK1-dependent induced pathway by SLAMF3 expression inhibits mitosis and control hepatocarcinoma cell proliferation. Oncotarget.

[92] Leslie EM, Deeley RG, Cole SP (2005). Multidrug resistance proteins: role of P-glycoprotein, MRP1, MRP2, and BCRP (ABCG2) in tissue defense. Toxicol Appl Pharmacol.

[93] Zhang YK, Wang YJ, Gupta P, Chen ZS (2015). Multidrug Resistance Proteins (MRPs) and Cancer Therapy. AAPS J.

[94] Fouquet G, Debuysscher V, Ouled-Haddou H, Eugenio MS, Demey B, Singh AR, Ossart C, Al Bagami M, Regimbeau JM, Nguyen-Khac E, Naassila M, Marcq I, Bouhlal H (2016). Hepatocyte SLAMF3 reduced specifically the multidrugs resistance protein MRP-1 and increases HCC cells sensitization to anti-cancer drugs. Oncotarget.

[95] McArdel SL, Terhorst C, Sharpe AH (2016). Roles of CD48 in regulating immunity and tolerance. Clin Immunol.

